# Perspectives on Racism in Health Care Among Black Veterans With Chronic Kidney Disease

**DOI:** 10.1001/jamanetworkopen.2022.11900

**Published:** 2022-05-12

**Authors:** Kevin A. Jenkins, Shimrit Keddem, Selamawite B. Bekele, Karisa E. Augustine, Judith A. Long

**Affiliations:** 1 Center for Health Equity Research and Promotion, Corporal Michael J. Crescenz Veterans Affairs Medical Center, Philadelphia, Pennsylvania; 2 Annenberg School of Communications, University of Pennsylvania, Philadelphia; 3 Perelman School of Medicine, University of Pennsylvania, Philadelphia; 4 Interpreting Attitudes Towards Minorities in Medicine (I AM) Research Group

## Abstract

**Importance:**

The burden of chronic kidney disease (CKD) and end-stage kidney disease falls disproportionately on Black individuals in the US, with Black veterans experiencing substantial consequences, and only a portion of the disparities in health conditions and health care can be explained by nonbiological factors. Among Black individuals, racism is likely one of those factors, suggesting the need to examine the consequences of racism and the resulting social structures that establish and perpetuate these racial disparities.

**Objective:**

To investigate the health care experiences of Black veterans with CKD and identify and explore the racial discrimination encountered by this vulnerable population.

**Design, Setting, and Participants:**

This qualitative study used semistructured interview guides to investigate the health care experiences of 36 Black veterans with CKD who received care at the Corporal Michael Crescenz Veterans Affairs Medical Center in Philadelphia, Pennsylvania, from October 2018 to September 2019. Interview transcripts were analyzed using applied thematic analysis.

**Results:**

Among 36 Black veterans with CKD who characterized racism in the context of their care at a Veterans Affairs medical center, the mean (SD) age was 66.0 (7.8) years; 35 participants (97.2%) were male, 1 participant (2.8%) was female, and 19 participants (52.8%) were married. The mean (SD) duration of military service was 8.0 (7.0) years. Overall, 15 participants (41.7%) were not dependent on dialysis, and hypertension was the most common comorbidity (9 participants [25.0%]). Veterans described the ways in which racism produced emotional and physical stress, including psychological symptoms (eg, anger and hurt) and physiological symptoms (eg, headaches). Veterans described a strong sense of distrust in the health care system coupled with a need to be hypervigilant during clinical encounters. When encountering racism, veterans described bottling up their feelings, which sometimes led to maladaptive behavior (eg, substance use). Veterans also described individual and collective positive strategies (eg, faith) for coping with the stress of racism.

**Conclusions and Relevance:**

In this study, Black veterans with CKD experienced racism in the clinical setting that produced physical and emotional stress and a strong sense of distrust in the health care system. These findings highlight an important opportunity for education and training of health care professionals in the implementation of trauma-informed approaches to care as a means of addressing race-based stress and trauma.

## Key Points

**Question:**

How do Black veterans with chronic kidney disease describe their experiences of racism in the health care setting?

**Findings:**

In this qualitative study of 36 Black veterans with chronic kidney disease, participants described feeling angry and resentful and experiencing stress as a result of encounters with racism; some veterans also expressed a strong sense of distrust in the health care system coupled with a need to be hypervigilant during clinical encounters. When encountering racism, veterans described using both negative and positive coping strategies.

**Meaning:**

These findings highlight an important opportunity for educating and training health care professionals in the implementation of trauma-informed approaches to care as a means of addressing race-based stress and trauma.

## Teaser

This qualitative study investigates the health care experiences and racial discrimination encountered by Black veterans with chronic kidney disease in the US.

## Introduction

The burden of chronic kidney disease (CKD) and end-stage kidney disease falls disproportionately on Black individuals in the US, with Black veterans experiencing substantial consequences. Compared with White veterans with CKD, Black veterans with CKD are twice as likely to progress to end-stage disease and make up approximately 37% of all patients with end-stage kidney disease in the US Department of Veterans Affairs (VA) health care system, while constituting only 12% of the veteran population.^1^ Limited access to high-quality health care, lower socioeconomic status (SES), exposure to environmental toxins, and health beliefs and behaviors are all associated with the racial disparities observed among Black individuals with CKD.^2,3,4^ Only a portion of disparities in health is associated with nonbiological factors.^5,6^ Among Black individuals in the US, racism is likely one of those factors, suggesting the need to examine racism and the resulting social structures that establish and perpetuate these racial disparities.

The impact of institutional racism is apparent in the association between low SES and CKD prevalence. Research has found that low SES over the life course is associated with 59% higher odds of CKD prevalence independent of demographic characteristics, insurance status, or the presence of other disease.^7^ Measured through educational level, employment status, income level, and poverty status, SES often reflects the manner in which structures and policies of institutional racism have kept Black people in lower SES compared with their White counterparts.^8^

Although Black veterans have access to health care through the Veterans Health Administration (VHA), every other aspect of their lives is affected by institutional racism. The interaction of SES and racism creates unfavorable social conditions that have consequences for the availability of resources to maintain health (eg, healthy food and safe places to exercise), access to health care (eg, transportation and proximity to clinical centers), and reduction of stressors (eg, financial worries, housing instability, and social support) among the Black population.^9,10^ In addition to institutionalized racism, individual discrimination affects the experiences of Black veterans.^11^ Black patients with CKD spend less time with health care professionals, experience worse treatment from clinicians, and feel that medical professionals do not convey necessary health care information, all while being referred, evaluated, and receiving kidney transplants less often than White patients.^1,12,13,14^

Despite increasing evidence of racial health disparities and health care inequalities in the US, research exploring the health care experiences of Black veterans with life-impeding chronic disease is limited. Understanding the experiences of marginalized populations can help to highlight why many efforts to address health disparities have been unsuccessful. This qualitative study investigated the health care experiences of Black veterans with CKD and identified and explored the racial discrimination encountered by this vulnerable population.

## Methods

### Study Sample

We conducted interviews with 36 Black veterans receiving care at Corporal Michael J. Crescenz VA Medical Center clinics in Philadelphia, Pennsylvania, from October 2018 to September 2019. All participants had a diagnosis of CKD and were (1) not dependent on dialysis (stage 3 [moderate; glomerular filtration rate, 30-59 mL/min/1.73 m^2^] or stage 4 [severe; glomerular filtration rate, 15-29 mL/min/1.73 m^2^] kidney disease), (2) dependent on dialysis (stage 5 [end-stage; glomerular filtration rate <15 mL/min/1.73 m^2^] kidney disease), or (3) posttransplant (stage 5 kidney disease). Interviews were conducted before or after appointments or during dialysis. This study was reviewed and approved by the institutional review board of the Corporal Michael J. Crescenz VA Medical Center. All participants provided oral informed consent to participate in the study and to be quoted directly at the beginning of their interview session. This study followed most of the items in the Consolidated Criteria for Reporting Qualitative Research (COREQ) reporting guideline for qualitative studies.

### Conceptual Framework

Our study was framed by an a priori conceptual framework using The House that Racism Built by Williams et al,^15^ which established 3 evidence-based pathways of racism that are directly associated with health and health care: cultural racism, institutional racism, and individual discrimination. According to Williams et al,^15^ individual discrimination can have consequences for health through psychological, biological, and behavioral factors; health care use; and individual and collective factors.

### Data Collection

Using purposeful sampling, we enrolled consecutive Black patients from each of the 3 CKD categories by approaching patients in person at the VA clinic between October 2018 and September 2019. Clinic nurses ascertained CKD diagnosis and stage through medical records reviews and referred patients for enrollment in the study. Race and sex were identified based on documentation in the electronic health record. Race recorded in the VHA electronic health record was based on self-identified race collected either during VA enrollment or an outpatient or inpatient encounter,^16^ in which veterans were asked to select any of the following races: American Indian or Alaska Native, Asian, Black, Native Hawaiian or Pacific Islander, or White. Data on ethnicity were not collected but were recorded when indicated. Sex as identified in the VHA electronic health record was based on sex assigned at birth.

All data were collected by a Black male principal investigator (K.A.J.) with a PhD in sociology and a Black male research assistant. Interviews lasting 30 to 60 minutes were conducted in person at the clinic and were audiorecorded and transcribed. Data were simultaneously collected and reviewed to assess thematic saturation; when saturation was achieved, no new interviews were conducted. The interview guide centered around 3 open-ended questions to understand veteran patients’ experiences of racism: (1) “What are some examples of racism you have experienced by a doctor?” (2) “What are some examples of racism you have experienced by someone who works at the hospital who is not your doctor?” and (3) “How do you think racism affects your health?”

### Statistical Analysis

Descriptive statistics were used to summarize demographic characteristics. Applied thematic analysis^17^ was used to guide the qualitative analysis process. Transcripts were cleaned by redacting identifying information and correcting typographical errors, then analyzed using NVivo qualitative analysis software, version 11 (QSR International). The codebook was created by the principal investigator along with an interdisciplinary research team (K.A.J., S.K., and J.A.L.) and included the following broad categories guided by the framework of Williams et al^15^ for understanding individual discrimination: psychological responses, biological responses, behavioral responses, health care use responses, and individual and collective responses. A team of 4 coders (all from the Interpreting Attitudes Towards Minorities in Medicine [I AM] Research Group) applied these codes to the transcripts by reading each line of text. Every fifth transcript (20% of the sample) was double coded by 2 coders to compare coding agreement. Agreement was measured using a κ coefficient, and coders met regularly to review coding and resolve discrepancies to produce a final mean κ coefficient of 0.98 (range, 0.60-1.00). The team (including K.A.J. and S.K.) reviewed the content of each category and identified the final themes.

## Results

Among 36 participants, the mean (SD) age was 66.0 (7.8) years; 35 participants (97.2%) were male, 1 participant (2.8%) was female, and 19 participants (52.8%) were married (Table). The mean (SD) duration of military service was 8.0 (7.0) years. Overall, 15 participants (41.7%) were not dependent on dialysis, and 10 participants (27.8%) reported having an immediate family member (eg, a parent or sibling) with CKD. Hypertension was the most common comorbidity (9 participants [25.0%]).

**Table.** Participant Demographic Characteristics

Characteristic	Participants, No. (%) (N = 36)
Age, mean (SD), y	66.0 (7.8)
Duration of military service, mean (SD), y	8.0 (7.0)
Sex assigned at birth	
Female	1 (2.8)
Male	35 (97.2)
Marital status	
Single	12 (33.3)
Married	19 (52.8)
Divorced	4 (11.1)
Widowed	1 (2.8)
CKD classification	
Non–dialysis-dependent (stages 3-4)^a^	15 (41.7)
Dialysis-dependent (stage 5)^b^	10 (27.8)
Posttransplant (stage 5)^b^	11 (30.6)
Immediate family member with CKD	10 (27.8)
Chronic conditions	
Diabetes	8 (22.2)
High blood pressure (hypertension)	9 (25.0)
Heart disease	3 (8.3)
HIV/AIDS	2 (5.6)
Agent Orange exposure	2 (5.6)
Stroke	1 (2.8)

Abbreviation: CKD, chronic kidney disease.

^a^ Stage 3 indicates moderate kidney disease (glomerular filtration rate, 30-59 mL/min/1.73 m^2^), and stage 4 CKD indicates severe kidney disease (glomerular filtration rate, 15-29 mL/min/1.73 m^2^).

^b^ Stage 5 indicates end-stage kidney disease (glomerular filtration rate <15 mL/min/1.73 m^2^).

Across all responses, we identified 4 themes: (1) association of racism with emotional and physical stress, (2) strong sense of distrust in the health care system coupled with a need to be hypervigilant, (3) bottling up of feelings, which sometimes led to maladaptive behavior, and (4) individual and collective positive strategies for coping with racism. Exemplary quotes are shown in the Box.

Box. **Sample Quotes From Participants**

**Theme 1: Association of Racism With Emotional and Physical Stress**

We’re under pressure all the time, it’s where the stress come in. Where I got to worry about—I’ll give you an example. A young Black man go to jail, he do 13 years in jail. Now he got a felon, but the Constitution says that by right, he paid his debt to society; 13 years you gave him for that, but yet, you stigmatized him with a felon. Now he come out, I can get a job, pay you taxes, but I can’t vote. So, the stresses that’s in my community, the stress that I feel? I’m stressing for my people. Damn. I hate to say that, but it makes my heart cry, man.
When I came back [from serving in the military], we was called a “nigger,” and they threw dog shit on us....And then, I had to get over that. And it’s like, hey, just stay in the house and do what you got to do. And then my [posttraumatic stress disorder] flared up on me and—so, I’m too old to go to jail. So, my surrounding is like this table, very small.
[Racism] makes me angry. It gives me headaches. It makes me irritable. It just—it makes me negative. It turns me into a negative person.
Because what’s balled up inside of me, brings stress not only on my kidneys but on every other part of my body. It’s stress on the head, it’s stress on my heart. I got sons that I wake up, and I thank God that they made it through our community, that adds on to all this pressure.
Then stress could be it, too—extra stress in life on an African American, coming up from a toddler on up. That could be the cause of [kidney disease], also.
I think it’ll give me high blood pressure. It’ll make me—by being high blood pressure, I mean, I’ll get angry over something that probably it should go my way but it’s not going my way because they underestimate my intelligence or something, you know. So, I believe that it do give me high blood pressure. It do probably make me run out here and eat something that I don’t want to eat because now I just wanna get away, you know.

**Theme 2: Distrust in the Health Care System and Hypervigilance**

That’s obvious. A poor Black man can’t go in a rich White man hospital and get the same treatment as a poor White man going in there with the same thing because they’re gonna take something that they have for White people and gonna want to tell me to go to the regular hospital.
I’ve watched certain things. I’ve watched how the nurses—for the most part at dialysis they all use gloves, but I just watch how—the interaction with other patients that are White. They may spend time with them, talking with them, this or that, less time with me or the other African Americans that are in the clinic. But that’s their people. And so I don’t hold them—I don’t blame them for treating their people good; I blame us for treating our people bad.
And it seemed like everything I asked her about—“Oh, don’t worry about that. Don’t worry.” I said, “What do you mean don’t worry about that? I asked you, I’m concerned about it.” “But I tell you when you need to worry.” So, I got rid of her. That one appointment I had with her, it was a lady, some Indian doctor. I got rid of her that same day because she didn’t show enough concern for my health care. She—you gotta listen to a patient in order to be able to treat that patient. She wasn’t willing to listen to me, so I seen we wasn’t gonna have a good relationship right there.
At that time, as a matter of fact, I felt like I was—I’m being a guinea pig on the medicine because they just issued it, didn’t check me out, didn’t see my—trying to get my—see my primary. They couldn’t get an appointment. Then they finally give me an appointment 3 or 4 months afterwards. So that had a lot to do with it. Then I tell them about it, then they say no, they denied it.
But I come here, and they treat me like every other patient, but then they still talk to you a certain kind of way until I tell them that I know a lot about what they’re talking about, what they’re doing. Or I want to look at the chart, and they’re like, “Why do you want to look at your chart? You’re not reading in it?” I say, “Yeah, I used to be a med tech, too. I know a lot of the lingo; I know some of the things.” And then it’s like—to them it’s almost like, wow. It’s a revelation as if—and it’s hard for me to understand why they would think that because in the military, everybody’s supposed to be the same.
Well, I feel the doctors, they might have a mindset, well, we brought whatever physical ailments we have on ourselves. And no matter what they tell us to do, we are not gonna listen to them, so we brought it on ourselves.

**Theme 3: Bottling Up of Feelings and Maladaptive Behavior**

I don’t—I try not to pay it any mind because, with me, really racism is like—I know I’m Black. I know a lot of people don’t like me because I’m Black. But after me serving in Vietnam and in Somalia, I don’t play the race card. I mean, I don’t even pay it no mind until it’s forced on me.
[Racism] don’t bother me; it goes in, in one ear and come out the other.
My sickness started a long time ago, when I first picked up that first alcohol, when I first picked up that first joint, when I took that first pill that wasn’t prescribe to me. They have to, in order to change the stress level we have today, we have to change behavior.
Interviewer: How do you usually handle stress? Participant: I go smoke a cigarette or drink a beer, or I just sit down to myself.
I change my behavior because I can’t react to everything. It’s gonna make you angry. And if you allow it to take away from your life that you’re spending on this earth, then that’s your fault.
[Racism] bothers me. It stresses me a little bit. But like I said, I try to keep that down because of my condition. I don’t need to be stressed out and lose this kidney.

**Theme 4: Positive Coping Strategies**

Well, that afflicts me because we’ve always been treated unfair, and it needs a lot of work. We need a lot of work on really turning ourselves around. I mean like, we have a lot of community involvement. I’m in a lot of community, different things with schools and all. I try to, you know, mentor young—I deal with the schools a lot in trying to mentor young people and all, to where, “pull your pants up, put a belt on.”
I find myself talking to my husband a lot because he gets angry. For instance, he was stopped by police officers, and they were really, really nasty to him and had my daughter in the car and scared my baby to death.
We all did our little family prayer around the table. And when I got to my part, I had so much to be thankful, by the time I got finished, everybody was crying. It was an ordeal, man. I think it paid off.
Every time things get kind of stressful and a little hard, and I talk to Jesus, and Jesus, he too wise to make a mistake. I ask him to order my steps. And I look at Jesus because he’s too wise to make a mistake.
I’ll be honest with you. I pray about it. That helps me to calm down. My main thing, I get—I have hypertension, and that really bothered me, and I was very irritable. And like I said, I had to calm down. So, I usually pray about it. And once I prayed about it, it really calmed me down quite a bit because my—it took my mind off what the problem was. So, that’s kind of what I deal with as far as stress is concerned.
I handle it by looking at sports, or just reading a book with my reader. Talk to my wife, my brother, or my son and talk to Jesus, my lord and my savior.

### Theme 1: Association of Racism With Emotional and Physical Stress

Participants’ psychological reactions to racism invoked a sense of hopelessness. Some participants spoke about a perpetual trauma that produced constant pressure and all-encompassing worry. As 1 participant explained, “We’re under pressure all the time.” This persistent pressure endured all day, every day, even when waking up in the morning. In addition, many participants portrayed the psychological result of racism as a feeling of deep anger and resentment derived from a sense of hurt and betrayal. One veteran said, “[Racism] makes me angry…It turns me into a negative person.” Veterans connected instances of racism to recurrences of posttraumatic stress disorder, which 1 veteran described as evoking the mentality experienced during “the war.”

When asked about the association between racism and health, most participants believed that racism had impacted their physical health, with high blood pressure and headaches reported as the most dominant physiological manifestations of racism. Accounts of the physical symptoms associated with racism referred directly to increased heart rate accompanied by a feeling of pent-up anger and energy. One veteran described the feeling as “stress on the head” and “stress on my heart,” and another stated, “I think it’ll give me high blood pressure.” This sensation, as described by 1 participant, “ate me up inside.” In their descriptions, the symptoms of CKD were associated with this stress and vice versa. Participants described these physical symptoms as being a lifelong battle from infancy to death, with 1 veteran explaining the struggle as “extra stress in life on an African American, coming up from a toddler on up.”

### Theme 2: Distrust in the Health Care System and Hypervigilance

When encountering racism in medical settings, participants articulated several reactions. They described feeling like they always had to prove themselves to medical staff and clinicians. They would arrive at their appointments worried about appearing presentable and perfect; even then, they described feeling judged and ignored. Participants described a strong sense of distrust in the medical establishment and the health care system. One veteran stated, “I’m being a guinea pig on the medicine because they just issued it, didn’t check me out.” In the dialysis clinic, they often felt they were being dismissed. Another described “being on guard” in medical settings. Veterans felt they needed to navigate these settings carefully and advocate for themselves (eg, by switching clinicians). One participant described switching clinicians after only 1 appointment because the clinician “didn’t show enough concern for my health care” and “wasn’t willing to listen to me.” At times, participants experienced racist incidents in waiting areas. In these instances, they felt the need to continue to be calm so they would not be perceived as the aggressor. Participants educated themselves and pushed back when their clinicians dismissed them. One veteran with previous experience as a medical technician said, “they [clinicians] still talk to you a certain kind of way until I tell them that I know a lot about what they’re talking about, what they’re doing.” Moreover, veterans filed complaints and moved their issues up the chain of command to enforce staff accountability for mistreatment. In some cases, veterans left the VA health care system or skipped appointments to avoid experiencing racism. Participants sometimes used humor and simply made light of the situation (eg, 1 participant stated, “I laugh at them”).

### Theme 3: Bottling Up of Feelings and Maladaptive Behavior

The immediate behavioral reactions to racism often began with ignoring the racist event. Participants stated that downplaying the initial encounter with discrimination and the subsequent stress helped to numb their ability to internalize their experience. They spoke about burying those feelings to preserve their sanity. To cope with the stress of racism in their lives and in the media, participants talked about turning to food for comfort.

One of the most insidious and pervasive maladaptive behaviors was the need to be hypervigilant to perceived social compliance, which included feeling the need to be perfect, always obey the law, and continually monitor their perceived demeanor. For example, 1 veteran said, “I change my behavior because I can’t react to everything,” and another stated, “in order to change the stress level we have today, we have to change behavior.” To handle the stress of racism, participants alluded to a need to stay secluded, especially when instances of racism (eg, police shootings and racist political narratives) attracted the attention of the national media. They were aware of their stress and felt a desire to control it, which they believed would mitigate the potential consequences of stress for their CKD. As 1 participant explained, “It stresses me a little bit…I try to keep that down because of my condition. I don’t need to be stressed out and lose this kidney.” Some veterans in our study described walking away from the indignity of individual racism in the moment, and some skipped health care appointments or switched clinicians. Maladaptive responses were rare, but participants also described experiencing impulses to drink alcohol or use other substances as a response to racism.

### Theme 4: Positive Coping Strategies

Participants described a variety of positive strategies for managing the stress associated with racism. Several participants described engaging in activism as an outlet for coping with stress. They discussed getting involved in the community, mentoring young adults, and wanting to influence and help the next generation. One veteran stated, “we’ve always been treated unfair, and it needs a lot of work…I try to, you know mentor the young.” These actions provided ways to keep busy and stay connected to their communities. By staying busy with clubs and organizations, they were able to generate support from their social networks.

Faith was another form of coping. Some participants talked about faith as a personal endeavor. When they experienced stress, they would “talk to Jesus.” Sometimes faith was a collective experience in the form of family prayer or church attendance. Faith was described as a way to manage stress and control thought patterns. As 1 veteran with hypertension explained, “once I prayed about it [the experience with racism], it really calmed me down quite a bit.”

Family was a fundamental source of support for participants. Spending time with grandchildren was a source of joy. Some participants described finding fulfillment in spending time at home with loved ones or taking care of family members who were ill or had disabilities. Talking and sharing stressful events with family was another major source of support. In addition, as veterans, they discussed spending time with friends from the military and the importance of that camaraderie for their well-being.

## Discussion

Black veterans who participated in this qualitative study characterized racism in the context of the care they received for CKD at a VA medical center. These veterans described the ways in which racism produced emotional and physical stress, including psychological symptoms (eg, anger and hurt) and physiological symptoms (eg, headaches). Participants also described a strong sense of distrust in the health care system coupled with a need to be hypervigilant during clinical encounters. When encountering racism, veterans recounted bottling up their feelings, which sometimes led to maladaptive behavior (eg, substance use). Veterans also described individual and collective positive strategies (eg, faith) for coping with racism.

Our findings that Black veterans with CKD experienced stress and strong distrust in the health care system may elucidate existing literature on discrimination, racism, and CKD. In the Healthy Aging in Neighborhoods of Diversity Across the Life Span study,^18^ researchers found that perceived racial discrimination was associated with worse kidney function. Another study of Black people at risk of CKD documented lower use of routine medical care among individuals with low trust in health care professionals.^19^ As expressed by participants in our study, perceived discrimination can lead to hopelessness, which has been found to lower self-efficacy,^20^ thereby impacting one’s ability to manage health. Our study was not the first to document discrimination within the VHA and highlight the importance of addressing race-based stress and trauma among veterans.^21^ Carlson et al^22^ developed a group-based intervention to target race-based stress and trauma through group therapy that provided a space for veterans from racial and ethnic minority groups to share and validate their experiences of discrimination.

In our study, Black veterans with CKD described using hypervigilance to overcome the health issues and health care consequences associated with racism. Participants felt an obligation and pressure to consistently present their best selves, yet they continued to experience dismissal of their concerns during encounters with health care professionals. Hypervigilance is a phenomenon associated with posttraumatic stress disorder and is highly prevalent among veterans of all races.^23^ Moreover, because service members live and work together and are bound by contractual terms of service while in the military, those from racial and ethnic minority groups may be more likely to experience chronic exposure to discrimination during their time in military service.^22^ Hypervigilance among racial minority groups has been associated with worse health outcomes,^23^ which may serve to corroborate the physical stress described by veterans in this study.

Racism and the adverse physical and mental health effects associated with experiencing racism represent a form of trauma,^24^ suggesting that trauma-informed care may be an approach to mitigate the adverse effects of racism in the care of Black veterans with CKD. Trauma-informed care, a standardized approach that aims to deliver sensitive care to patients who have experienced a variety of traumatic experiences, including rape, child abuse, and intimate partner violence, can guide clinicians’ interactions with patients in a way that is culturally appropriate and patient-centered and that harnesses adaptive coping strategies.^25,26^ An important component of trauma-informed care is avoiding retraumatization. Because health care environments can be a source of retraumatization, it is important that health care professionals and systems create health care settings that patients perceive as safe and inclusive.^25^

Trauma-informed care provides clinicians with a platform for acknowledging the intersection of trauma and racism.^27^ The Substance Abuse and Mental Health Services Administration has developed guidance for the use of trauma-informed care in mental health settings that can be extended to other clinical settings.^28^ Guidance from the Substance Abuse and Mental Health Services Administration emphasizes developing a shared understanding of the impact of trauma, using trauma screening, developing policies to respond to trauma individually and institutionally, and avoiding retraumatization.^27,28^ To implement a trauma-informed approach that successfully screens and responds to patients’ experiences of discrimination, it is important to educate health care professionals about the nonbiological structural factors associated with racism. This framework, called structural competency,^29,30^ was developed to foster an understanding of how factors such as racism impact health and are associated with health disparities and how those factors can have implications for the clinical encounter.

During the clinical encounter, clinicians can use a number of strategies to deliver trauma-informed care. First, clinicians can be aware of bidirectional culture^31^ and use cultural humility,^32^ recognizing that one cannot fully understand another’s culture and having the humility to learn from patients and self-reflect by acknowledging the power imbalances and implicit biases that exist.^32^ Clinicians can establish a partnership of mutual respect, articulating to the patient that they will work together to ensure the treatment plan is convenient and feasible. Clinicians can educate their patients about both diagnosis and treatment, using language that is clear while making sure to acknowledge the perspectives of the patient in front of them.^31^

Trauma-informed approaches require regular and ongoing training as well as quality improvement and preparation of the entire multidisciplinary team to incorporate trauma-informed responses into daily practice.^25,33^ Continuous quality improvement encourages all health care team members to continuously ask, “How are we doing?” and “What can we do?”^34^ This improvement could be achieved in several different ways. First, annual assessments of a patient’s experience with racism, especially with regard to the health care the patient is receiving, would guide the clinical delivery of care for the individual and help to establish a continual process improvement approach. Second, encouragement of feedback from patients and clinicians, combined with a committee of patients, family, and health care professionals that regularly reviews complaints, concerns, and suggestions, would help to ensure patients’ voices are heard, respected, and incorporated into care delivery improvements. As with quality improvement efforts, these efforts would need to be seen as nonjudgmental and targeted at improving care so that all individuals involved feel comfortable speaking up without fear of retribution. Third, the formation of oversight groups to conduct internal biannual assessments and review care processes and outcomes by race would help to ensure the focus remains on providing equitable care.

### Limitations

This study has several limitations. First, the study primarily included male veterans, who represent most of the veteran population with CKD. Second, the participants recruited for this study use the VHA for their health care; thus, their experiences may be different from those of Black veterans receiving care outside the VHA. Third, data were only collected at the Corporal Michael J. Crescenzi VA Medical Center in Philadelphia, Pennsylvania. Therefore, the experiences described by veterans in this study may not be representative of veterans’ experiences at facilities in more rural settings in other geographic locations. Fourth, interview questions assume that participants experienced racism and that racism is associated with health. Nevertheless, participants frequently expressed opinions with similar themes that fit well into the domains of the framework of Williams et al,^15^ which suggests that the findings of this study may be applicable to a broader patient population.

## Conclusions

In this qualitative study, Black veterans with CKD described health care experiences that were retraumatizing and further worsened their psychological and physical responses to racism, potentially exacerbating CKD symptoms. Implementing care models that acknowledge racism as traumatic experience is one way the VA and other health care institutions can lead the nation in developing antiracist health care.
