# Error in Quotation

**DOI:** 10.1001/jamanetworkopen.2022.20419

**Published:** 2022-06-10

In the Original Investigation titled “Perspectives on Racism in Health Care Among Black Veterans With Chronic Kidney Disease,”^1^ published May 12, 2022, one participant quotation included potentially identifiable information about a physician. This article has been corrected.^1^
